# The Non-Anhydrous, Minimally Basic Synthesis of the Dopamine D_2_ Agonist [18F]MCL-524

**DOI:** 10.3390/chemistry3030075

**Published:** 2021-09-09

**Authors:** James A. H. Inkster, Anna W. Sromek, Vamsidhar Akurathi, John L. Neumeyer, Alan B. Packard

**Affiliations:** 1 Division of Nuclear Medicine and Molecular Imaging, Boston Children’s Hospital, 300 Longwood Ave., Boston, MA 02115, USA;; 2 Harvard Medical School, 25 Shattuck St., Boston, MA 02115, USA;; 3 Division of Basic Neuroscience, McLean Hospital, 115 Mill St., Belmont, MA 02478, USA

**Keywords:** fluorine-18, aporphines, dopamine D_2_ agonist, high-affinity state, PET

## Abstract

The dopamine D_2_ agonist MCL-524 is selective for the D_2_ receptor in the high-affinity state (D_2_^high^), and, therefore, the PET analogue, [^18^F]MCL-524, may facilitate the elucidation of the role of D_2_^high^ in disorders such as schizophrenia. However, the previously reported synthesis of [^18^F]MCL-524 proved difficult to replicate and was lacking experimental details. We therefore developed a new synthesis of [^18^F]MCL-524 using a “non-anhydrous, minimally basic” (NAMB) approach. In this method, [^18^F]F^−^ is eluted from a small (10–12 mg) trap-and-release column with tetraethylammonium tosylate (2.37 mg) in 7:3 MeCN:H_2_O (0.1 mL), rather than the basic carbonate or bicarbonate solution that is most often used for [^18^F]F^−^ recovery. The tosylated precursor (1 mg) in 0.9 mL anhydrous acetonitrile was added directly to the eluate, without azeotropic drying, and the solution was heated (150 °C/15 min). The catechol was then deprotected with the Lewis acid In(OTf)_3_ (10 equiv.; 150 °C/20 min). In contrast to deprotection with protic acids, Lewis-acid-based deprotection facilitated the efficient removal of byproducts by HPLC and eliminated the need for SPE extraction prior to HPLC purification. Using the NAMB approach, [^18^F]MCL-524 was obtained in 5–9% RCY (decay-corrected, *n* = 3), confirming the utility of this improved method for the multistep synthesis of [^18^F]MCL-524 and suggesting that it may applicable to the synthesis of other ^18^F-labeled radiotracers.

## 1. Introduction

### 1.1. Aporphines

**Figure 1.**
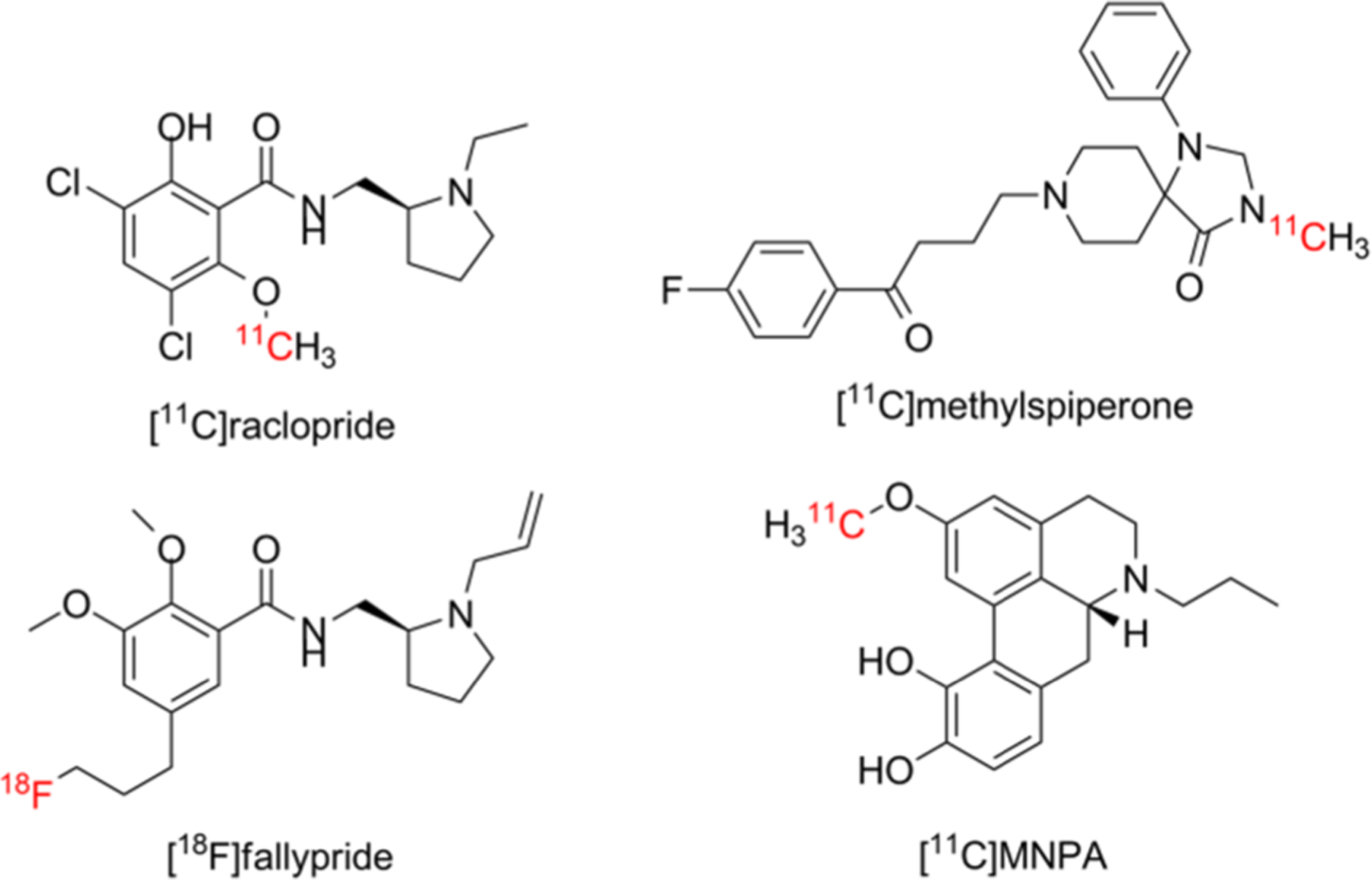
Selected PET radioligands of the dopamine D_2_ receptor.

Dopamine D_2_ receptor dysfunction is a hallmark of several neurological diseases and disorders, including schizophrenia, Parkinson’s disease, Tourette’s syndrome and addiction [1–14]. Like other G-protein-coupled receptors, D_2_ transitions between two states that vary in their affinity for endogenous dopamine: a high-affinity, or functional, state (D_2_^high^), and a low-affinity state (D_2_^low^) [15–18]. Thus, PET (positron emission tomography) radiopharmaceuticals that can be used to measure differences in the population of D_2_^high^ versus D_2_^low^ may be useful in improving the understanding and diagnosis of these diseases [19–22]. However, most PET probes of postsynaptic dopamine receptor function evaluated to date suffer from limitations that preclude their use for this application. For example, [^11^C]raclopride (Figure 1), a D_2_/D_3_ ligand [23,24], has a high affinity for D_2_-like receptors in striata, but exhibits reduced D_2_ binding during periods of high synaptic dopamine concentration [25–27]. The structurally related benzamide, [^18^F]fallypride (Figure 1), also does not distinguish between D_2_ and D_3_ [28], and the butyrophenone, [^11^C]methylspiperone (Figure 1), is not selective for D_2_ versus 5HT_2_ receptors [29]. More importantly, all of these radioligands are D_2_ antagonists, which do not distinguish between the high- and low-affinity states of the D_2_ receptor.

**Scheme 1.**
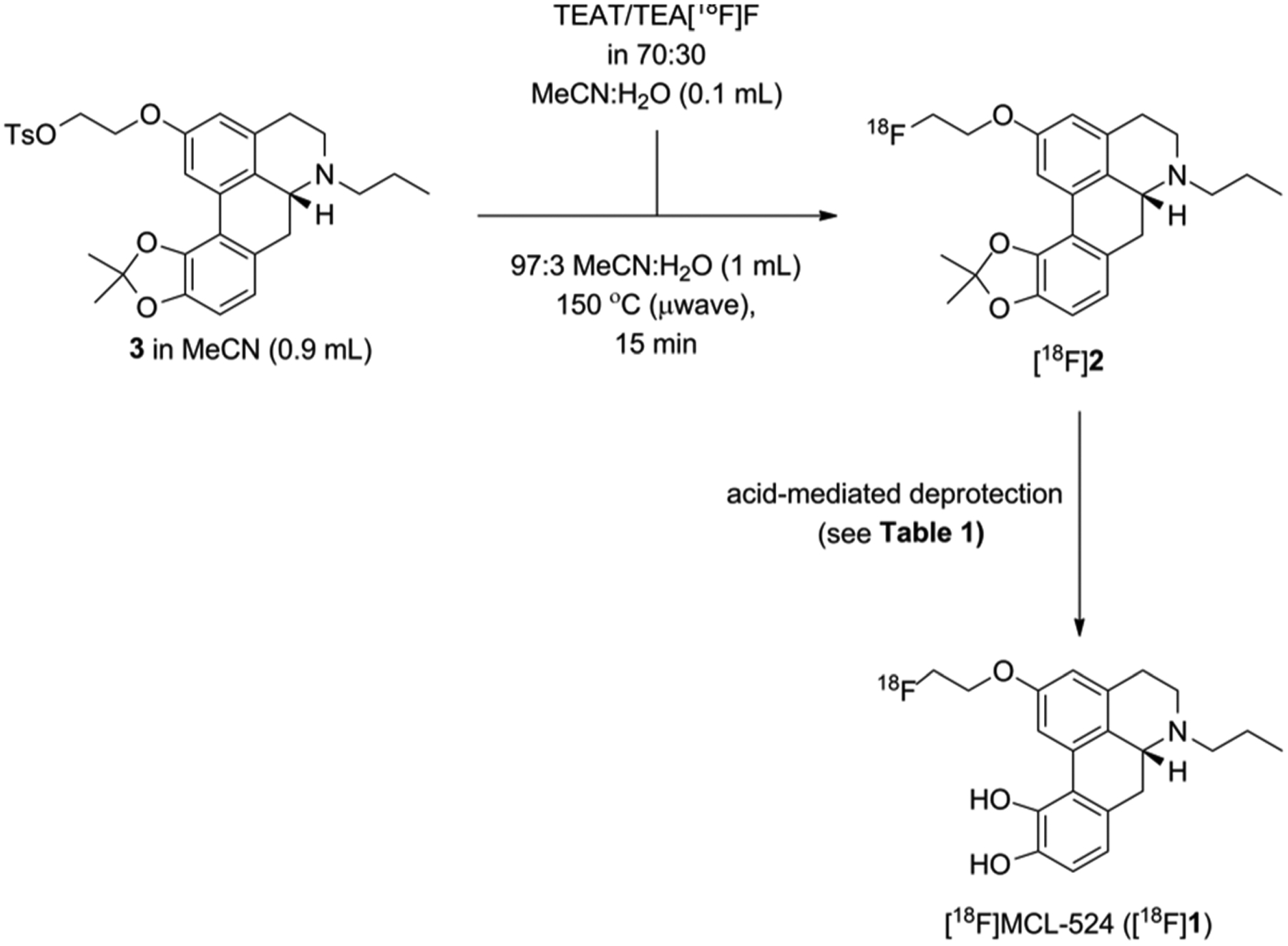
NAMB synthesis of [^18^F]MCL-524 ([^18^F]**1**).

Aporphines are a class of dopamine D_2_ receptor agonists derived from apomorphine. As agonists, aporphines exhibit a higher affinity for the D_2_^high^ state than for the D_2low_ state [11,15,16,30–32]; and therefore, it has been proposed that radiolabeled aporphines may be useful for probing dopamine supersensitivity and other phenomena associated with changes in the D_2_^high^/D_2low_ ratio [7–10,14,17,21]. To this end, a variety of non-radioactive fluorinated aporphines were synthesized and evaluated versus [^3^H]domperidone using competitive binding assays in rat striatal homogenates, and MCL-524 (**1**; Scheme 1) was identified as a candidate radiotracer owing to its high affinity for D_2_^high^ (*K*_i_ = 3.7 ± 1.2 nM) and much lower affinity for D_2low_ (*K*i = 990 ± 35 nM) [32]. An ^18^F-labeled analogue was subsequently prepared, its specificity was compared to that of [^11^C]MNPA (Figure 1), and its pharmacokinetics were evaluated in cynomolgus monkeys [33]. Notably, the mean striatal non-displaceable binding potential (BP_ND_) value was 2.0 for [^18^F]MCL-524 versus 1.4 for [^11^C]MNPA, a 1.5-fold increase. [^18^F]MCL-524 BP_ND_ values were reduced by 89% and 56% in response to acute raclopride and D-amphetamine pretreatment, respectively, and it exhibited excellent radiotracer kinetics, showing a rapid accumulation in the striatum and a high striatum:cerebellum ratio within an hour after administration.

However, the previously reported synthesis of [^18^F]MCL-524 has several limitations, including a low synthetic yield and the use of 6 M HCl to deprotect the catechol post-labeling, which degrades the product and complicates product purification. In order to address these limitations, we sought a new approach to this synthesis that obviated these limitations.

### 1.2. NAMB ^18^F Chemistry

Fluorine-18 (t_1/2_ = 109.8 min) is the “gold standard” for many PET imaging applications; however, the radiolabeling of potential targeting vectors with [^18^F]F^−^ is often challenging and sometimes impossible in light of the high temperatures and basic conditions that are typically employed (K_2_CO_3_ or KHCO_3_, Kryptofix 2.2.2, MeCN or DMSO, 70–200 °C). Furthermore, it is generally accepted that radiolabeling compounds with [^18^F]F^−^ require scrupulously anhydrous reaction conditions. This requirement has resulted in the widespread use of the azeotropic drying of the [^18^F]F^−^ after it is extracted from the irradiated [^18^O]H_2_O target by anion-exchange (AEX) chromatography and prior to its use in any synthetic procedure. In recent years, however, several laboratories have proposed methods to simplify or eliminate these [^18^F]F^−^ preparation steps [34–39].

In addition to eliminating the [^18^F]F^−^ “drydown” step, we also introduced the use of non-basic tetraalkylammonium salt solutions for the extraction of [^18^F]F- from the AEX “trap-and-release” column [40–42], a method later used by others [43–45]. This “non-anhydrous, minimally basic” (NAMB) strategy involves the use of a small AEX column to extract [^18^F]F^−^ from [^18^O]H_2_O, from which it is then efficiently eluted using an aqueous solution of tetraethylammonium tosylate (TEATos) and diluted with a solution of the precursor compound in anhydrous DMSO or MeCN [46]. The key to the success of this approach is the small size of the AEX column (10–12 mg), which can be eluted with as little as 100 μL of 7:3 MeCN:H_2_O. This small elution volume permits dilution to a final reaction volume (1 mL) that maintains an effective precursor concentration and is compatible with automated synthesis systems while keeping the water concentration at ≤5%. This “damp” reaction matrix can be heated directly, without the need for azeotropic drying. In addition to increasing the overall efficiency by reducing the number of radiosynthetic steps, this approach: (a) avoids losses in radioactivity associated with the volatilization of H[^18^F]F and absorption of activity on the surface of the reaction vessel during dry-down; (b) is suitable for both nucleophilic aromatic and nucleophilic aliphatic ^18^F-fluorinations; and (c) is compatible with the volumes of aqueous [^18^F]F^−^ obtained from standard cyclotron targets (1–3 mL). The NAMB approach was previously used to synthesize [^18^F]fluorobenzaldehyde and [^18^F]fallypride [46], demonstrating that at least some [^18^F]F^−^ incorporation reactions do not require anhydrous basic conditions in order to proceed efficiently.

### 1.3. Preperation of [^18^F]MCL-524

The previously reported synthesis of [^18^F]MCL-524 utilized a standard nucleophilic aliphatic ^18^F-fluorination reaction (DMSO, 150–160 °C, 10 min) to generate intermediate [^18^F]**2** (Scheme 1) from tosylated precursor MCL-556 (**3**; Scheme 1), followed by the removal of the acetonide protecting group with 6 M HCl at 90–110 °C (10 min) [33]. Notably, neither the efficiency of the initial ^18^F-fluorination step, the efficiency of the catechol deprotection step nor the final isolated radiochemical yield (RCY) were reported. Upon attempting to replicate this work, we observed a significant accumulation of chemical by-products after treatment with HCl and low conversion of [^18^F]**2** to [^18^F]**1**. We hypothesized that, if the ^18^F-fluorination step were conducted in an environment free of the carbonate base, which is typically present in standard [^18^F]F^−^ incorporation reactions, an efficient and reproducible acid-mediated deprotection would be easier to achieve and might allow for the removal of acetonide under milder conditions. Herein, we describe an alternative radiosynthesis of [^18^F]MCL-524 using NAMB [^18^F]F^−^ incorporation chemistry, as well as an unconventional Lewis-acid-based method for the deprotection of intermediate [^18^F]**2**.

## 2. Materials and Methods

### Radiosynthesis of [^18^F]MCL-524 ([^18^F]**1**) via In(OTf)_3_-Mediated Deprotection

An aliquot of [^18^F]F^−^ (190 MBq) in [^18^O]H_2_O was diluted to 2 mL with H_2_O and the [^18^F]F^−^ was captured on a commercial QMA AEX column (carbonate form, 10–12 mg; Med-Chem Imaging, LLC), which was previously activated with water (1 mL). After washing the column with anhydrous MeCN (3 mL), a continuous flow of Ar was passed through the column for 10 min. The [^18^F]F^−^ was eluted from the column in the reverse direction into a microwavable test tube using a 100 μL solution of TEATos (23.7 mg/mL) in 7:3 MeCN:H_2_O. Residual eluent was ejected from the column using a syringe filled with air (10 mL). The precursor, MCL-556 (**3**; 1 mg), in anhydrous MeCN (900 μL), was added to the eluate, and the tube was crimp-sealed, magnetically stirred for 20 s and heated to 150 °C in a microwave heater (Biotage^®^ Initiator+) for 15 min. After removing small aliquots for radio-TLC (10% EtOH in CH_2_Cl_2_, silica gel, 49 ± 2% radiochemical conversion (RCC), *n* = 4) and analytical HPLC (HPLC 1, Program A in the Supporting Information), the ^18^F-fluorination reaction mixture containing [^18^F]**2** was added to solid In(OTf)_3_ (10.35 mg) in a microwave test tube. Water was added (100 μL), and the reaction mixture was heated to 150 °C (microwave) for 20 min. The percent conversion of intermediate [^18^F]**2** to [^18^F]**1** (38%) was assayed by analytical HPLC (HPLC 1, Program A). The crude reaction mixture was diluted to 2 mL with sodium acetate buffer (50 mM AcOH/2.5 mM NaOAc) containing 0.1 mg/mL ascorbic acid and purified by preparative HPLC (HPLC 2, Program D). The collected product was diluted with water (50 mL) and trapped on a Sep-Pak^®^ C18 Plus cartridge, which was previously activated with 3 mL of EtOH and 10 mL of water. [^18^F]MCL-524 was eluted from the column with EtOH (1.5 mL) and diluted with 0.9% saline (1.5 mL) containing sodium ascorbate (3 mg/mL). The final formulation was passed through a 0.2 μm filter to afford 7.06 MBq (191 μCi) of [^18^F]MCL-524 [3.7% non-decay corrected, 9.2% decay corrected (DC)] in 1:1 EtOH:sodium ascorbate (4.5 mg) in isotonic saline (3 mL total). Product identity and molar activity were assessed by HPLC (HPLC 1, Program C). Total synthesis time was 146 min from start-of-synthesis.

## 3. Results

### 3.1. Summary of Non-Radioactive Synthesis

**Scheme 2.**
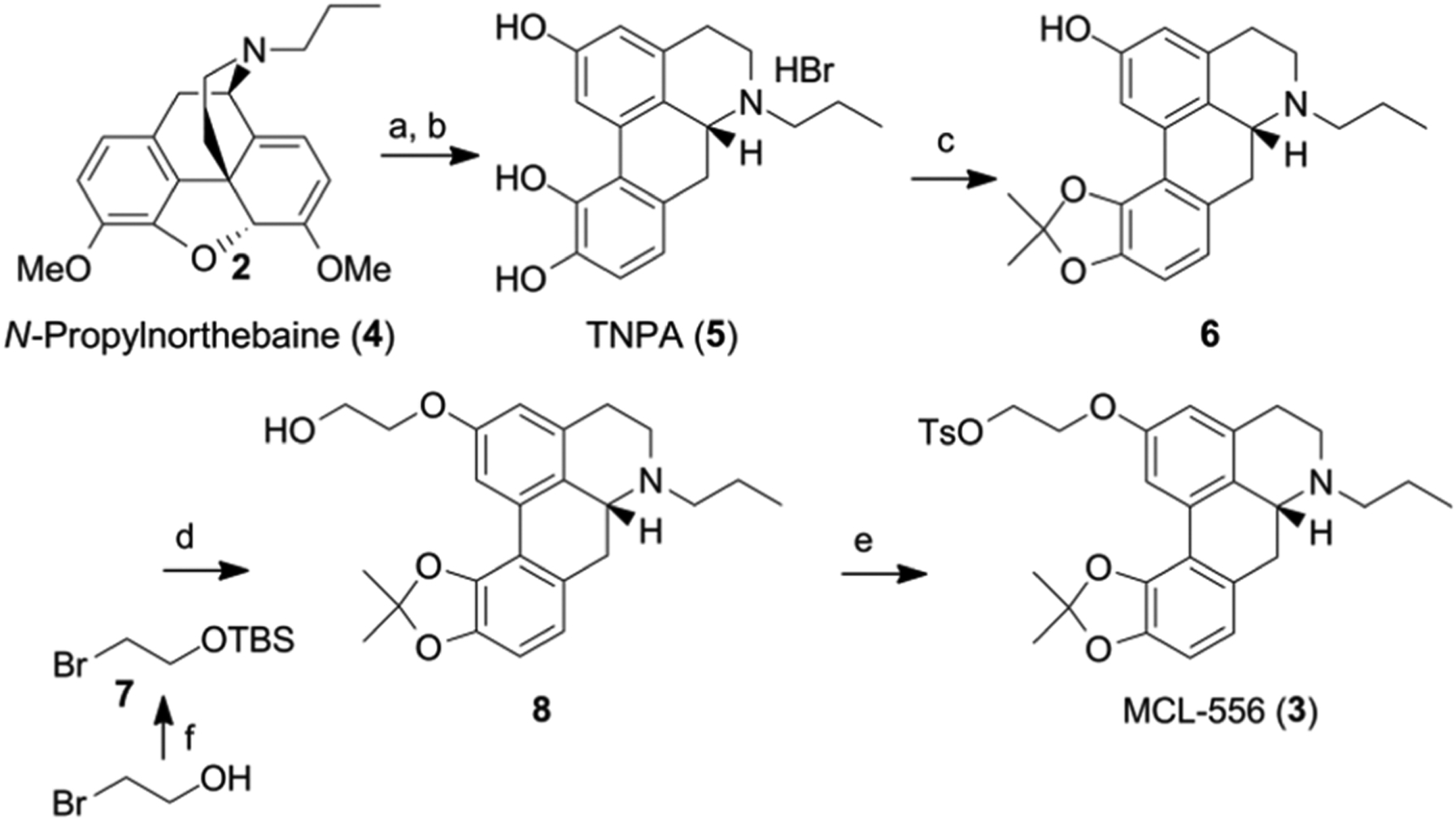
Synthesis of precursor MCL-556 (**3**). Reagents and conditions: (a) *i*. DIAD, MeCN, heat; *ii*. MeOH, EtOAc; (b) 48% HBr/AcOH, 120 °C; (c) CH_3_COCH_3_, P_2_O_5_, THF, 60 °C; (d) **7**, 5 N NaOH, THF, 70 °C; (e) Ts_2_O, Et_3_N, DMAP, CH_2_Cl_2_; (f) TBSCl, Et_3_N, CH_2_Cl_2_.

Tosylated precursor MCL-556 (**3**) [33] was synthesized as previously described, with several minor modifications (Scheme 2 and Scheme S1 in the Supporting Information). Thebaine was *N*-demethylated with DIAD [47], and then alkylated to afford *R*-(−)-N-n-propylnorthebaine (**4**) according to the previously published procedure [48]. Intermediate **4** was then *O*-demethylated by refluxing in 48% HBr in glacial acetic acid to afford R-(−)-2,10,11-trihydroxy-n-propylnorapomorphine hydrobromide (**5**; TNPA) [48]. The catechol moiety of **5** was protected as an acetonide using acetone and phosphorus pentoxide in THF, as previously described [49]. The acetonide-protected intermediate (**6**) was then alkylated with TBS-protected 1-bromoethan-2-ol (**7**) under phase transfer conditions in order to afford the 2-hydroxyethoxy aporphine (**8**), which was tosylated to afford the radiosynthetic precursor **3**. Nonradioactive MCL-524 was synthesized from the common intermediate **4** following previously reported procedures (Scheme S2 in Supporting Information) [32,50].

### 3.2. Summary of Radioactive Synthesis

**Figure 2.**
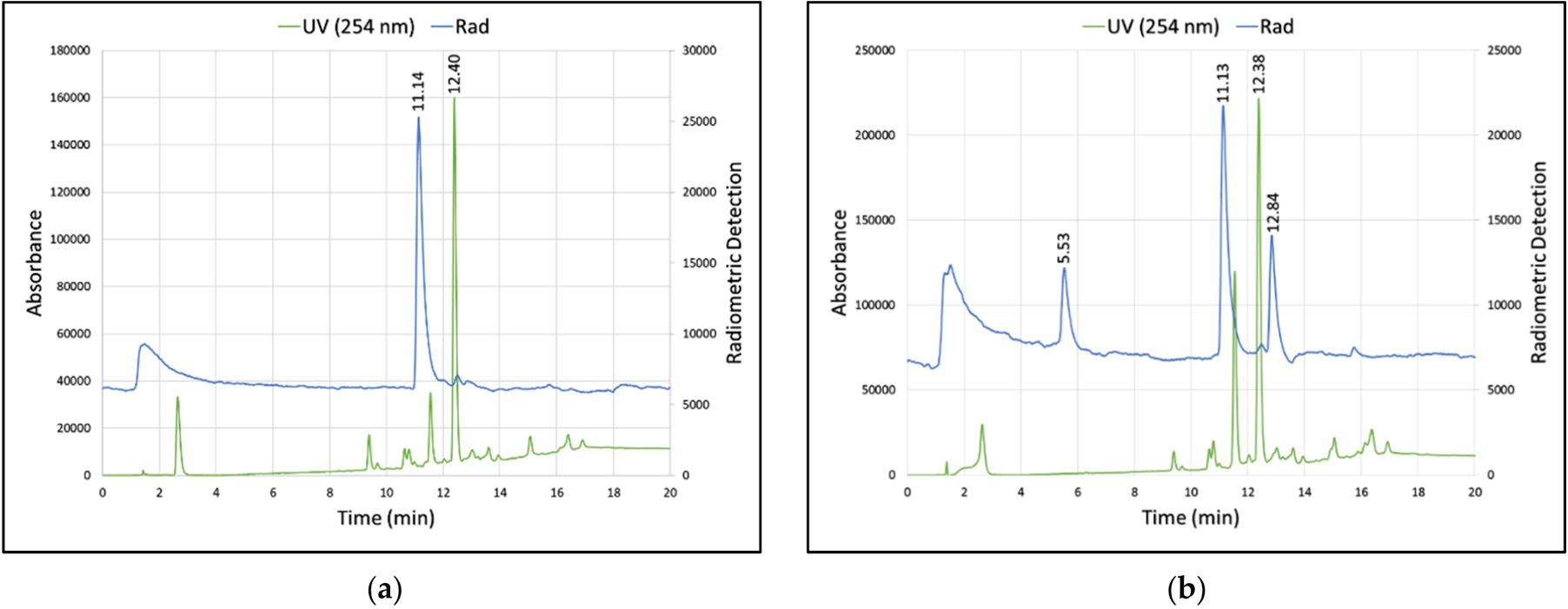
Analytical radio-HPLCs of two [^18^F]**2** (t_R_ = 11.14 min) reaction mixtures (TEATos, 97% MeCN, 150 °C, 15 min). In both traces, starting material **3** can be seen at ~12.4 min (254 nm). Whereas reaction (**a**) contains only one significant radiochemical product, reaction (**b**) contains radio-impurities at 5.5 and 12.8 min. The peak at 12.8 min co-eluted with toluenesulfonyl fluoride standard. See the Supporting Information for HPLC conditions (HPLC 1, Program A).

[^18^F]Fluoride trap-and-release and radio-fluorination conditions were similar to those used for the NAMB preparation of [^18^F]fallypride [46], using a solvent matrix consisting of TEATos in 97% MeCN (Scheme 1). Upon microwave heating (150 °C, 15 min), the ^18^F-fluorination of **3** (1 mg) in 97% MeCN (1 mL) produced acetonide-protected [^18^F]**2** in a RCC of 51 ± 4% (radio-TLC, *n* = 6, Figure S1 in the Supporting Information). Under these conditions, the principal radiochemical product was [^18^F]**2** (Figure 2a); however, some reaction mixtures contained significant radio-impurities, observed at ~5.5 min and ~12.8 min using analytical HPLC (Figure 2b). Interestingly, a peak at 5.5 min was also sometimes observed after the NAMB preparation of an unrelated radiotracer under identical HPLC conditions [46], albeit in much smaller quantities. The 12.8 min peak was determined to be toluenesulfonyl [^18^F]fluoride based on its retention time versus the non-radioactive standard. The highly variable appearance of these radiochemical by-products suggests that they are the result of small batch-to-batch changes in the reagent concentration and/or water content.

**Figure 3.**
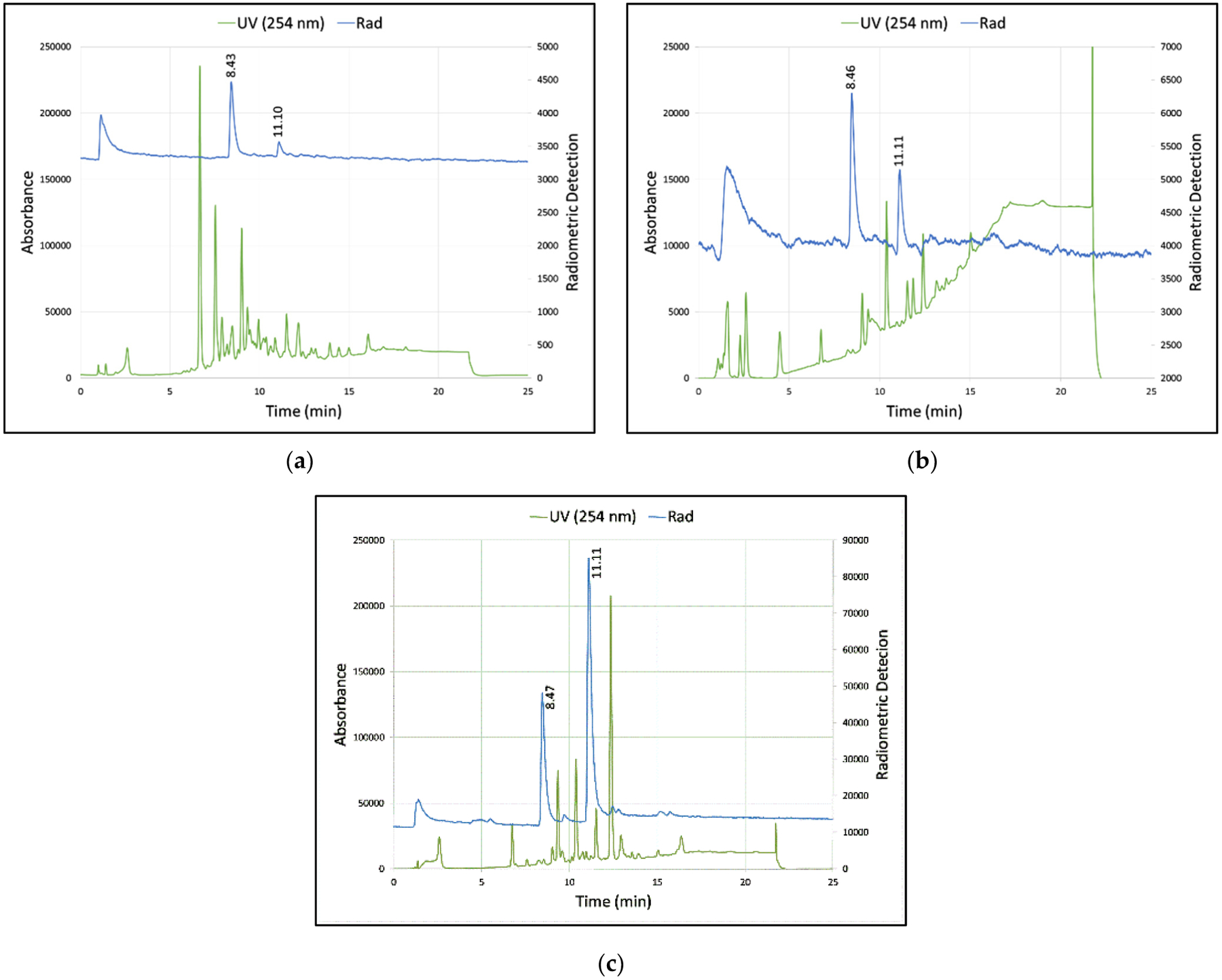
Representative radio-HPLCs of catechol deprotection reactions using: (**a**) 6 N H_2_SO_4_ (Table 1, Entry 2); (**b**) 90% TFA (Table 1, Entry (4); (**c**) In(OTf)_3_ (Table 1, Entry (3)). [^18^F]2 = ~11.1 min. [^18^F]1 = ~8.4 min. See the Supporting Information for HPLC conditions (HPLC 1, Program A).

After NAMB ^18^F fluorination of the tosylated precursor **3**, the treatment of the crude reaction mixture with 1:1 6 N H_2_SO_4_:MeOH for 5 min at 100 °C resulted in a 5% conversion of intermediate [^18^F]**2** to [^18^F]MCL-524 ([^18^F]**1**), as determined by HPLC (Table 1, Entry 1). Increasing the time (10 min) and temperature (150 °C) led to a marked increase in percent conversion (86%; Table 1, Entry 2). However, the use of these conditions also significantly increased the production of chemical impurities that we anticipated would complicate the isolation of the final product by HPLC (Figure 3a). Alternative acidic deprotection conditions were therefore explored, with the extent of conversion from intermediate [^18^F]**2** to final product [^18^F]**1** measured by HPLC. Deprotection with 90% TFA for 5 min at 100 °C led to a 67% conversion and resulted in a crude reaction mixture with fewer by-products than with 6 N H_2_SO_4_:MeOH (Figure 3b), but required the product to be separated from the acidic reaction mixture by SPE prior to HPLC purification. The isolated DC-RCY of [^18^F]MCL-524 under these conditions was 8% after the HPLC purification, concentration by C18 SPE and final formulation in 10% EtOH in isotonic saline. When the deprotection reaction time was extended to 10 min, the conversion yield was 83% and DC isolated yield was 14%.

Alternatively, the deprotection of [^18^F]**2** using an excess of the Lewis acid In(OTf)_3_ [51] (10 molar equivalents relative to **3**) produced only a 33–38% conversion (*n* = 3), even at high temperatures (150 °C) and long reaction times (20 min) (Figure 3c). However, unlike deprotection using protic acids (e.g., 6 N H_2_SO_4_, 90% TFA), these reaction mixtures were significantly less acidic (pH = 3–4) and thus did not require SPE of the crude mixture prior to HPLC purification. Similar to treatment with TFA, and unlike treatment with H_2_SO_4_, the distribution of non-radioactive by-products after In(OTf)_3_ deprotection did not impede the purification of [^18^F]**1** by semi-preparative HPLC. [^18^F]MCL-524 prepared via In(OTf)_3_ deprotection was obtained in a greater than 98% radiochemical purity and an overall radiochemical yield of 5–9% DC (*n* = 3) over 146–199 min. The product identity was verified by the co-injection of [^18^F]**1** with the non-radioactive standard (Figure S2, Supporting Information).

In keeping with the limitations of our research facility, most of the radiochemical experiments reported here employed relatively small aliquots of [^18^F]F^−^ (197–255 MBq), resulting in modest molar activities (Table 1) and final product quantities. However, one experiment was carried out that started with 715 MBq of [^18^F]F^−^, and this reaction yielded 47 MBq of [^18^F]MCL-524, enough for preclinical PET imaging and providing evidence that this method can be scaled to accommodate the larger quantities of [^18^F]F^−^ employed in clinical production sites. In the context of developing a receptor-targeting radiopharmaceutical, this is an important result because starting with larger quantities of [^18^F]F^−^ will also increase the molar activity of the final product.

The results summarized in Table 1 suggest that 90% TFA is superior to In(OTf)_3_ in terms of catechol deprotection. However, the increased deprotection efficiency observed using 90% TFA at 100 °C (5 or 10 min) did not result in substantially higher isolated RCYs of [^18^F]**1**. It is likely that the improved deprotection yield obtained with 90% TFA (83% conversion over 10 min) is largely offset by the transfer and trapping losses associated with the additional SPE step required to extract the radiotracer from the concentrated acid reaction mixture prior to HPLC purification. Another Lewis acid, Yb(OTf)_3_, was found to be ineffective for catechol deprotection under conditions identical to those employed with In(OTf)_3_ (Table 1, Entry 6).

## 4. Discussion

Non-standard [^18^F]F^−^ extraction and labeling conditions were used to prepare [^18^F]MCL-524, a D_2_ agonist that demonstrates a higher affinity activated state of the receptor. ^18^F-fluorination reactions utilized tetraethylammonium tosylate as both the eluent to remove the [^18^F]F^−^ from trap-and-release AEX columns and as the phase-transfer catalyst in reaction mixtures containing 97:3 MeCN:H_2_O. Despite using non-anhydrous reaction conditions and relatively low masses of precursor (1 mg), the [^18^F]F^−^ incorporation was >50%. This work provides further evidence that non-basic tetraalkylammonium salts and non-anhydrous reaction conditions may lead to improvements in ^18^F labeling protocols, which demand speed and operational simplicity for success.

The removal of the acetonide protecting group by treatment with the Lewis acid In(OTf)_3_ in the presence of water produced overall yields of [^18^F]MCL-524 similar to those obtained with 90% TFA, and both of these approaches yielded reaction mixtures that permitted the isolation of a chemically pure product by HPLC. However, in contrast to solutions containing concentrated protic acids, the In(OTf)_3_ reaction mixture can be directly injected onto a semi-preparative HPLC column, eliminating the need for a solid-phase extraction prior to injection, and improving overall synthetic efficiency. This study suggests that Lewis acids may be an attractive alternative to protic acids for the syntheses of other ^18^F-labeled compounds that might benefit from a milder reaction condition and/or simplified purification procedures.

## Supplementary Material

Inkster et al Chemistry 2021 SI

## Figures and Tables

**Table 1.** Methods for acid-mediated deprotection of [^18^F]**2**.

Entry	Reagent Added (Volume)	Time (min)	Temp (°C)	Conversion (%)^a^	DC-RCY (%)	Molar Activity^b^ (GBq/μmol)
1	1:1 6 N H_2_SO_4_:MeOH (0.2 mL)	5	100	5	-^c^	-
2	1:1 6 N H_2_SO_4_:MeOH (0.2 mL)	10	150	86	-^c^	-
3	In(OTf)_3_^d^ in H_2_O (0.1 mL)	20	150	33, 35, 38^e^	6, 5, 9^e^	0.9, 0.7, 0.3^e^
4	90% TFA (1 mL)	5	100	67	8	1.6
5	90% TFA (1 mL)	10	100	83	14	2.0
6	Yb(OTf)_3_^d^·xH_2_O in H_2_O (0.1 mL)	20	150	0	-	

a Assessed by HPLC.

b Based on a mass standard curve.

c [^18^F]MCL-524 not isolated by HPLC due to the presence of convoluting chemical by-products.

d 10 equivalents.

e Individual experiments.

## References

[1] Beaulieu JM; Gainetdinov RR The physiology, signaling, and pharmacology of dopamine receptors. Pharmacol. Rev 2011, 63, 182–217. 21303898 10.1124/pr.110.002642

[2] Blum K; Sheridan PJ; Wood RC; Braverman ER; Chen TJ; Cull JG; Comings DE The D2 dopamine receptor gene as a determinant of reward deficiency syndrome. J. R. Soc. Med 1996, 89, 396–400. 8774539 10.1177/014107689608900711 PMC1295855

[3] Noble EP The DRD2 gene in psychiatric and neurological disorders and its phenotypes. Pharmacogenomics 2000, 1, 309–333. 11256581 10.1517/14622416.1.3.309

[4] Volkow ND; Morales M The Brain on Drugs: From Reward to Addiction. Cell 2015, 162, 712–725. 26276628 10.1016/j.cell.2015.07.046

[5] Volkow ND; Wang GJ; Fowler JS; Tomasi D; Telang F Addiction: Beyond dopamine reward circuitry. Proc. Natl. Acad. Sci. USA 2011, 108, 15037–15042. 21402948 10.1073/pnas.1010654108 PMC3174598

[6] Volkow ND; Fowler JS; Wang GJ; Baler R; Telang F Imaging dopamine’s role in drug abuse and addiction. Neuropharmacology 2009, 56 (Suppl. 1), 3–8. 18617195 10.1016/j.neuropharm.2008.05.022 PMC2696819

[7] Seeman MV; Seeman P Is schizophrenia a dopamine supersensitivity psychotic reaction? Prog. Neuropsychopharmacol. Biol. Psychiatry 2014, 48, 155–160. 24128684 10.1016/j.pnpbp.2013.10.003 PMC3858317

[8] Seeman P Are dopamine D2 receptors out of control in psychosis? Prog. Neuropsychopharmacol. Biol. Psychiatry 2013, 46, 146–152. 23880595 10.1016/j.pnpbp.2013.07.006

[9] Seeman P Schizophrenia and dopamine receptors. Eur. Neuropsychopharmacol 2013, 23, 999–1009. 23860356 10.1016/j.euroneuro.2013.06.005

[10] Seeman P All roads to schizophrenia lead to dopamine supersensitivity and elevated dopamine D2(high) receptors. CNS Neurosci. Ther 2011, 17, 118–132. 20560996 10.1111/j.1755-5949.2010.00162.x PMC6493870

[11] Seeman P Antiparkinson therapeutic potencies correlate with their affinities at dopamine D2(High) receptors. Synapse 2007, 61, 1013–1018. 17853435 10.1002/syn.20453

[12] Seeman P; Schwarz J; Chen JF; Szechtman H; Perreault M; McKnight GS; Roder JC; Quirion R; Boksa P; Srivastava LK; Psychosis pathways converge via D2high dopamine receptors. Synapse 2006, 60, 319–346. 16786561 10.1002/syn.20303

[13] Seeman P Targeting the dopamine D2 receptor in schizophrenia. Expert Opin. Ther. Targets 2006, 10, 515–531. 16848689 10.1517/14728222.10.4.515

[14] Nakata Y; Kanahara N; Iyo M Dopamine supersensitivity psychosis in schizophrenia: Concepts and implications in clinical practice. J. Psychopharmacol 2017, 31, 1511–1518. 28925317 10.1177/0269881117728428

[15] Sibley DR; De Lean A; Creese I Anterior pituitary dopamine receptors. Demonstration of interconvertible high and low affinity states of the D-2 dopamine receptor. J. Biol. Chem 1982, 257, 6351–6361. 6176582

[16] Sibley DR; Creese I Regulation of ligand binding to pituitary D-2 dopaminergic receptors. Effects of divalent cations and functional group modification. J. Biol. Chem 1983, 258, 4957–4965. 6833284

[17] Seeman P; Weinshenker D; Quirion R; Srivastava LK; Bhardwaj SK; Grandy DK; Premont RT; Sotnikova TD; Boksa P; El-Ghundi M; Dopamine supersensitivity correlates with D2High states, implying many paths to psychosis. Proc. Natl. Acad. Sci. USA 2005, 102, 3513–3518. 15716360 10.1073/pnas.0409766102 PMC548961

[18] Seeman P; Tallerico T; Ko F Dopamine displaces [^3^H]domperidone from high-affinity sites of the dopamine D2 receptor, but not [^3^H]raclopride or [^3^H]spiperone in isotonic medium: Implications for human positron emission tomography. Synapse 2003, 49, 209–215. 12827639 10.1002/syn.10232

[19] Skinbjerg M; Sibley DR; Javitch JA; Abi-Dargham A Imaging the high-affinity state of the dopamine D2 receptor in vivo: Fact or fiction? Biochem. Pharmacol 2012, 83, 193–198. 21945484 10.1016/j.bcp.2011.09.008 PMC3610415

[20] Shalgunov V; van Waarde A; Booij J; Michel MC; Dierckx R; Elsinga PH Hunting for the high-affinity state of G-protein-coupled receptors with agonist tracers: Theoretical and practical considerations for positron emission tomography imaging. Med. Res. Rev 2019, 39, 1014–1052. 30450619 10.1002/med.21552 PMC6587759

[21] van Wieringen JP; Booij J; Shalgunov V; Elsinga P; Michel MC Agonist high- and low-affinity states of dopamine D(2) receptors: Methods of detection and clinical implications. Naunyn Schmiedebergs Arch. Pharmacol 2013, 386, 135–154. 23224422 10.1007/s00210-012-0817-0

[22] Zhang L; Villalobos A Strategies to facilitate the discovery of novel CNS PET ligands. EJNMMI Radiopharm. Chem 2017, 1, 13. 29564389 10.1186/s41181-016-0016-2 PMC5843814

[23] Sokoloff P; Giros B; Martres MP; Bouthenet ML; Schwartz JC Molecular cloning and characterization of a novel dopamine receptor (D3) as a target for neuroleptics. Nature 1990, 347, 146–151. 1975644 10.1038/347146a0

[24] Breier A; Su TP; Saunders R; Carson RE; Kolachana BS; De Bartolomeis A; Weinberger DR; Weisenfeld N; Malhotra AK; Eckelman WC; Schizophrenia is Associated with Elevated Amphetamine-Induced Synaptic Dopamine Concentrations: Evidence from a Novel Positron Emission Tomography Method. Proc. Natl. Acad. Sci. USA 1997, 94, 2569–2574. 9122236 10.1073/pnas.94.6.2569 PMC20129

[25] Narendran R; Hwang DR; Slifstein M; Hwang Y; Huang Y; Ekelund J; Guillin O; Scher E; Martinez D; Laruelle M Measurement of the proportion of D2 receptors configured in state of high affinity for agonists in vivo: A positron emission tomography study using [^11^C]N-propyl-norapomorphine and [^11^C]raclopride in baboons. J. Pharmacol. Exp. Ther 2005, 315, 80–90. 16014571 10.1124/jpet.105.090068

[26] Volkow ND; Wang GJ; Fowler JS; Logan J; Schlyer D; Hitzemann R; Lieberman J; Angrist B; Pappas N; MacGregor R; Imaging endogenous dopamine competition with [11C]raclopride in the human brain. Synapse 1994, 16, 255–262. 8059335 10.1002/syn.890160402

[27] Dewey SL; Smith GS; Logan J; Brodie JD; Fowler JS; Wolf AP Striatal binding of the PET ligand ^11^C-raclopride is altered by drugs that modify synaptic dopamine levels. Synapse 1993, 13, 350–356. 8480281 10.1002/syn.890130407

[28] Mukherjee J; Yang ZY; Brown T; Lew R; Wernick M; Ouyang X; Yasillo N; Chen CT; Mintzer R; Cooper M Preliminary assessment of extrastriatal dopamine D-2 receptor binding in the rodent and nonhuman primate brains using the high affinity radioligand, ^18^F-fallypride. Nucl. Med. Biol 1999, 26, 519–527. 10473190 10.1016/s0969-8051(99)00012-8

[29] Hartvig P; Eckernas SA; Ekblom B; Lindstrom L; Lundqvist H; Axelsson S; Fasth KJ; Gullberg P; Langstrom B Receptor binding and selectivity of three ^11^C-labelled dopamine receptor antagonists in the brain of rhesus monkeys studied with positron emission tomography. Acta Neurol. Scand 1988, 77, 314–321. 3260438 10.1111/j.1600-0404.1988.tb05915.x

[30] Finnema SJ; Seneca N; Farde L; Shchukin E; Sovago J; Gulyas B; Wikstrom HV; Innis RB; Neumeyer JL; Halldin C A preliminary PET evaluation of the new dopamine D-2 receptor agonist C-11 MNPA in cynomolgus monkey. Nucl. Med. Biol 2005, 32, 353–360. 15878504 10.1016/j.nucmedbio.2005.01.007

[31] Subburaju S; Sromek AW; Seeman P; Neumeyer JL New Dopamine D2 Receptor Agonist, [^3^H]MCL-536, for Detecting Dopamine D2high Receptors in Vivo. ACS Chem. Neurosci 2018, 9, 1283–1289. 29641175 10.1021/acschemneuro.8b00096 PMC8412031

[32] Sromek AW; Si YG; Zhang T; George SR; Seeman P; Neumeyer JL Synthesis and Evaluation of Fluorinated Aporphines: Potential Positron Emission Tomography Ligands for D2 Receptors. ACS Med. Chem. Lett 2011, 2, 189–194. 21666830 10.1021/ml1001689 PMC3110009

[33] Finnema SJ; Stepanov V; Nakao R; Sromek AW; Zhang T; Neumeyer JL; George SR; Seeman P; Stabin MG; Jonsson C; ^18^F-MCL-524, an ^18^F-Labeled Dopamine D2 and D3 Receptor Agonist Sensitive to Dopamine: A Preliminary PET Study. J. Nucl. Med 2014, 55, 1164–1170. 24790219 10.2967/jnumed.113.133876 PMC4587887

[34] Lee SJ; Oh SJ; Chi DY; Moon DH; Ryu JS High Yielding F-18 Fluorination Method by Fine Control of the Base. B Kor. Chem. Soc 2012, 33, 2177–2180.

[35] Sergeev ME; Morgia F; Lazari M; Wang C Jr.; van Dam RM Titania-catalyzed radiofluorination of tosylated precursors in highly aqueous medium. J. Am. Chem. Soc 2015, 137, 5686–5694. 25860121 10.1021/jacs.5b02659 PMC4485375

[36] Richarz R; Krapf P; Zarrad F; Urusova EA; Neumaier B; Zlatopolskiy BD Neither azeotropic drying, nor base nor other additives: A minimalist approach to ^18^F-labeling. Org. Biomol. Chem 2014, 12, 8094–8099. 25190038 10.1039/c4ob01336k

[37] Basuli F; Zhang X; Woodroofe CC; Jagoda EM; Choyke PL; Swenson RE Fast indirect fluorine-18 labeling of protein/peptide using the useful 6-fluoronicotinic acid-2,3,5,6-tetrafluorophenyl prosthetic group: A method comparable to direct fluorination. J. Labelled Comp. Radiopharm 2017, 60, 168–175. 27990672 10.1002/jlcr.3487 PMC5344719

[38] Kniess T; Laube M; Steinbach J “Hydrous ^18^F-fluoroethylation”—Leaving off the azeotropic drying. Appl. Radiat. Isot 2017, 127, 260–268. 28688368 10.1016/j.apradiso.2017.06.010

[39] Kwon YD; Son J; Yun MJ; Chun JH Azeotropic drying-free aliphatic radiofluorination to produce PET radiotracers in a mixed organic solvent system. Tetrahedron Lett. 2018, 59, 2848–2852.

[40] Inkster JAH; Akurathi V; Sromek AW; Chen Y; Neumeyer JL; Packard AB A new radiosynthesisof the D2 agonist [^18^F]MCL-524 using tetraethylammonium tosylateas a phase transfer catalyst and In(OTf)_3_ for catechol deprotection. In Proceedings of the 22nd International Symposium on Radiopharmaceutical Sciences, Dresden, Germany, 14–19 May 2017.

[41] Inkster JAH; Akurathi V; Sromek AW; Neumeyer JL; Packard AB Tetraethylammoniumperchlorate and tosylate as non-basic anion exchange reagents and 1^8F^-fluorinationphase-transfer catalysts: Application to the synthesis of [^18^F]fallypride. In Proceedings of the 22nd International Symposium on Radiopharmaceutical Sciences, Dresden, Germany, 14–19 May 2017.

[42] Inkster J; Akurathi V; Chen Y; Sromek A; Neumeyer J; Packard A ^18^F chemistry without azeotropic distillations: Tetraethylammonium salts as combined anion exchange reagents and phase transfer catalysts. J. Nucl. Med 2016, 57, 328.

[43] Orlovskaya V; Antuganov D; Fedorova O; Timofeev V; Krasikova R Tetrabutylammonium tosylate as inert phase-transfer catalyst: The key to high efficiency S(N)2 radiofluorinations. Appl. Radiat. Isot 2020, 163, 109195. 32561038 10.1016/j.apradiso.2020.109195

[44] Orlovskaya V; Fedorova O; Nadporojskii M; Krasikova R A fully automated azeotropic drying free synthesis of O-(2-[^18^F]fluoroethyl)-l-tyrosine ([^18^F]FET) using tetrabutylammonium tosylate. Appl. Radiat. Isot 2019, 152, 135–139. 31299450 10.1016/j.apradiso.2019.07.006

[45] Fedorova O; Orlovskaya V; Nadporojskii M; Krasikova R Automated synthesis of the 16 alpha- F-18 fluoroestradiol (F-18 FES): Minimization of precursor amount and resulting benefits. Radiochim. Acta 2020, 108, 979–988.

[46] Inkster JAH; Akurathi V; Sromek AW; Chen Y; Neumeyer JL; Packard AB A non-anhydrous, minimally basic protocol for the simplification of nucleophilic ^18^F-fluorination chemistry. Sci. Rep 2020, 10, 6818. 32321927 10.1038/s41598-020-61845-y PMC7176689

[47] Kim K; Kim K Synthesis of the new thebaine derivatives by the Diels-Alder reaction with northebaine. Taehan Hwahakhoe Chi 1988, 32, 371–376.

[48] Gao YG; Baldessarini RJ; Kula NS; Neumeyer JL Synthesis and dopamine receptor affinities of enantiomers of 2-substituted apomorphines and their N-n-propyl analogues. J. Med. Chem 1990, 33, 1800–1805. 1971309 10.1021/jm00168a040

[49] Steiger C; Finnema SJ; Raus L; Schou M; Nakao R; Suzuki K; Pike VW; Wikstroem HV; Halldin C A two-step one-pot radiosynthesis of the potent dopamine D2/D3 agonist PET radioligand [^11^C]MNPA. J. Labelled Compd. Radiopharm 2009, 52, 158–165.

[50] Si YG; Gardner MP; Tarazi FI; Baldessarini RJ; Neumeyer JL Synthesis and binding studies of 2-O- and 11-O-substituted N-alkylnoraporphines. Bioorg. Med. Chem. Lett 2008, 18, 3971–3973. 18585036 10.1016/j.bmcl.2008.06.016

[51] Golden KC; Gregg BT; Quinn JF Mild, versatile, and chemoselective indium(III) triflate-catalyzed deprotection of acetonides under microwave heating conditions. Tetrahedron Lett. 2010, 51, 4010–4013.

