# Supplementary material for: The Non-Anhydrous, Minimally Basic Synthesis of the Dopamine D2 Agonist [18F]MCL-524
Source: Chemistry (Basel). Author manuscript; Available in PMC 2023 Oct 12. (PMC10569134; doi:10.3390/chemistry3030075)
Supplement: Inkster et al Chemistry 2021 SI [file NIHMS1935113-supplement-Inkster_et_al_Chemistry_2021_SI.pdf]

Supporting Information

“Non-anhydrous, minimally basic” synthesis of the dopamine D2 agonist [^{18}F]MCL-524

James A. H. Inkster, Anna W. Sromek, Vamsi Akurathi, John L. Neumeyer and Alan B. Packard

DOI: 10.3390/chemistry3030075

Table of Contents

General Information 3

Scheme S1: Synthesis of non-radioactive precursor 3 4

Details related to the synthesis of precursor 3 4

Scheme S2: Synthesis of MCL-524 (1) 6

Details related to the synthesis of MCL-524 (1) 6

Figure S1: Representative radio-TLC of intermediate [^{18}F]2 7

Figure S2: Co-injection of [^{18}F]MCL-524 with ^{19}F standard 7

References 8

General Information

Chemicals and Media

Unless otherwise noted, all reagents and solvents were purchased from Acros Organics (Fair Lawn, NJ), Alfa Aesar (Ward Hill, MA), Fisher Scientific (Hampton, NH), Oakwood Chemical (Estill, SC), or Millipore-Sigma (St. Louis, MO), and were used without further purification. Morphine and thebaine were supplied by Mallinckrodt Pharmaceuticals (St. Louis, MO). Silica gel (40-63 µm) for flash chromatography was obtained from Silicycle (Quebec City, Canada). MP-1 and QMA anion-exchange cartridges (both carbonate form) for $[^{18}F]F^-$ separation were obtained from MedChem Imaging (Boston, USA). Sep-Pak® C18 and tC18 solid-phase extraction cartridges were obtained from Waters (Milford, MA).

Thin Layer Chromatography (TLC)

TLC was performed using pre-coated silica gel 60 F_{254} aluminum sheets from EMD Millipore (Billerica, MA). Non-radioactive compounds were visualized under ultraviolet light at 254 nm. A Fujifilm BAS-5000 Phosphor Imager with Multi Gauge v3.0 software was used to visualize ^{18}F.

Liquid Chromatography

The HPLC systems described below, including data acquisition modules, are Shimadzu Prominence. All gamma detectors were optimized for 511 keV photons.

HPLC 1. Analytical. Pump: LC-20AD. Diode array detector (DAD): SPD-M20A. Radiation detector: Harshaw NaI(Tl) detector with Canberra NIM electronics. For low-resolution liquid chromatography-mass spectroscopy (LC-MS), an Advion expression^S CMS apparatus was added in-line.

HPLC 2. For manual preparations. Pump: LC-20AT. UV/Vis detector: SPD-20A. Radiation detector: Carroll & Ramsey Model 105S.

Program A. Analytical. Column: EMD Millipore Purosphere® RP-18 endcapped, 5 µm, 4 mm × 125 mm. Solvent system: gradient elution, 10% MeCN in H_2O, both containing 0.1% trifluoroacetic acid (TFA), for 2 min, raised to 90% MeCN in H_2O containing 0.1 % TFA over 13 min, hold for 5 min, flow rate = 1 mL/min. Detector: 190-800 nm (DAD).

Program C. Analytical. Column: EMD Millipore Purosphere® RP-18 endcapped, 5 µm, 4 mm × 125 mm. Solvent system: isocratic elution, 25:75 MeCN:H_2O, both containing 0.1% TFA, flow rate = 1 mL/min. Detector: 190-800 nm (DAD).

Program D. Semi-preparative. Column: ES Industries Chromegabond WR C18, 5 µm, 120 Å, 9.6 mm × 250 mm. Solvent system: isocratic elution, 50:50 AcOH (50 mM)/NaOAc (2.5 mM) with 0.1 mg/mL ascorbic acid:MeCN, flow rate = 3 mL/min. Detector: 254 nm.

Program E. Analytical. Column: EMD Millipore Purosphere® RP-18 endcapped, 5 µm, 4 mm × 125 mm. Solvent system: gradient elution, 10% MeCN in H_2O, both containing 0.1% formic acid, for 2 min, raised to 90% MeCN in H_2O containing 0.1 % formic acid over 13 min, hold for 5 min, flow rate = 1 mL/min. Detector: 190-800 nm (DAD).

NMR

^1H and ^{13}C NMR spectra were recorded with either a Varian 400-MR or Varian Mercury 300 spectrometer (Palo Alto, CA). NMR solvents were obtained from Cambridge Isotope Laboratories (Andover, MA). Chemical shifts (δ) are reported in ppm relative to the hydrogenated residue of the deuterated solvents.

High-resolution Mass Spectroscopy (HR-MS)

HR-MS were obtained by the Mass Spectrometry Lab at the University of Illinois at Urbana-Champaign (Urbana, IL).

Elemental Microanalysis (EA)

EA was carried out by Atlantic Microlab, Inc. (Norcross, GA).

Microwave

Microwave heating of radiochemical reactions was carried out using a Biotage® Initiator+ apparatus. The reactions were carried out using crimped glass reaction vessels (0.5-2 mL size) containing magnetic stir vanes.

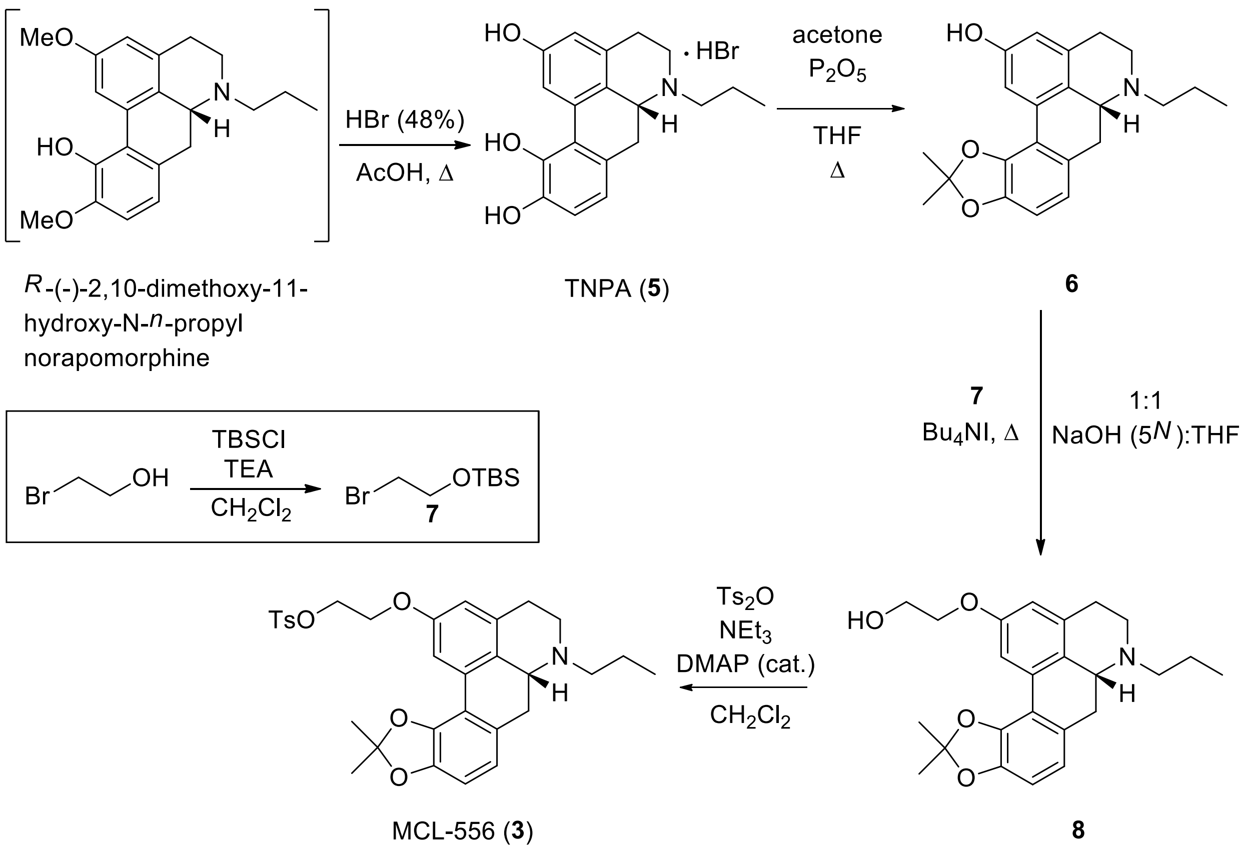

Scheme S1. Synthesis of non-radioactive precursor 3.

Details related to the synthesis of precursor 3

2,10,11-trihydroxy-N-n-propyl-norapomorphine (TNPA; 5), hydrobromide salt

TNPA (HBr salt) was prepared in four synthetic steps from thebaine, as described in Kim *et al.*[1], Si *et al.*[2] and Gao *et al.*[3] Overall yield: 71%. M.P.= 204-206 ºC (lit. 203-206 ºC).[3]

^{1}H NMR (400 MHz, CD_3OD) δ 7.92 (d, *J* = 2.5 Hz, 1H), 6.67 (q, *J* = 8.0 Hz, 2H), 6.54 (d, *J* = 2.5 Hz, 1H), 4.02 (dd, *J* = 13.9, 3.8 Hz, 1H), 3.87 – 3.74 (m, 1H), 3.56 (ddd, *J* = 13.1, 11.5, 5.2 Hz, 1H), 3.35 – 3.23 (m, 3H), 3.23 – 3.08 (m, 1H), 2.93 (d, *J* = 14.6 Hz, 1H), 2.78 – 2.64 (m, 1H), 1.99 – 1.71 (m, 2H), 1.09 (t, *J* = 7.3 Hz, 3H).

^{13}C NMR (101 MHz, CD_3OD) δ 156.88, 144.82, 143.25, 134.18, 130.56, 124.33, 119.54, 119.31, 118.72, 115.13, 113.92, 112.75, 61.21, 55.32, 49.13, 31.61, 25.98, 17.08, 10.04.

LC-MS (HPLC 1, Program E): 312 m/z $[M+H]^+$, 100%. 353 m/z $[M+H+CH_3CN]^+$, 11%.

(-)-2-hydroxy-N-n-propyl-10,11-propylidenenorapomorphine (6)

Compound **5** was prepared as described in Steiger *et al.*,[4] with minor modifications. To a 500-mL round-bottom flask were added TNPA (HBr salt, 4 g, 10.2 mmol), THF (112 mL), dry acetone (50 mL), and P_2O_5 (5 g). The contents were stirred and refluxed for 2 h. An additional equal portion of P_2O_5 and acetone were added, and the reaction mixture refluxed for another 4 h. The next day, the cooled reaction mixture was concentrated, and the residue was basified with NH_4OH solution and extracted with CH_2Cl_2 (3 × 200 mL). The combined organic extracts were washed with brine, dried over sodium sulfate, filtered, and

concentrated. The residue was purified by silica gel column chromatography using a 1:20 to 1:10 to 1:5 MeOH to CH_2Cl_2 gradient to afford 854 mg (24%) of acetonide-protected aporphine **6** as a brown foam.

^{1}H NMR (300 MHz, Chloroform-*d*) δ 7.40 (d, *J* = 2.6 Hz, 1H), 6.72 – 6.64 (m, 1H), 6.59 (dd, *J* = 7.8, 1.0 Hz, 1H), 6.46 (d, *J* = 2.5 Hz, 1H), 3.44 (d, *J* = 6.8 Hz, 1H), 3.21 (dd, *J* = 11.1, 5.6 Hz, 1H), 3.10 (dt, *J* = 9.7, 6.1 Hz, 2H), 2.93 (td, *J* = 11.4, 10.3, 5.9 Hz, 1H), 2.58 (ddd, *J* = 32.0, 13.4, 6.6 Hz, 4H), 1.75 (s, 3H), 1.64 (s, 3H), 1.73 – 1.51 (m, 2H), 0.96 (t, *J* = 7.3Hz, 3H).

^{13}C NMR (75 MHz, Chloroform-*d*) δ 154.36, 146.70, 143.90, 134.72, 132.10, 128.91, 120.00, 117.84, 117.03, 114.28, 112.36, 106.90, 77.20, 59.40, 56.09, 49.29, 33.99, 29.03, 26.12, 25.92, 18.80, 12.07.

LC-MS (HPLC 1, Program E): 352 m/z $[M+H]^+$, 100%. 393 m/z $[M+H+CH_3CN]^+$, 60%.

1-bromoethyl-2-(t-butyldimethyl)silyloxyethane (7)

To a 20 mL vial were added: TBSCl (1 M in CH_2Cl_2; 6.4 mL) and bromoethanol (0.40 mL), and the solution was stirred. To the resulting yellow solution was slowly added dropwise triethylamine (0.90 mL); the solution immediately became colorless upon addition of the first drop. The solution was stirred at RT overnight; a white precipitate formed. The suspension was diluted with hexanes (50 mL) and washed with water (50 mL) and brine (50 mL). The organic phase was dried over Na_2SO_4, filtered, and concentrated to afford **7** in quantitative yield (339 mg). This oil was deemed analytically pure by NMR.

^{1}H NMR (400 MHz, Chloroform-*d*) δ 3.89 (t, *J* = 6.5 Hz, 1H), 3.39 (t, *J* = 6.5 Hz, 1H), 0.90 (s, 5H), 0.09 (s, 3H).

^{13}C NMR (101 MHz, Chloroform-*d*) δ 63.51, 33.25, 25.81 (3C), -5.29 (2C).

EA: calcd: C, 40.17, H, 8.01; found: C, 40.11, H, 8.04

(-)-2-hydroxyethoxy-N-n-propyl-10,11-propylidenenorapomorphine (8)

The following synthesis was first reported in Finnema *et al.;* [5] however, the NMR, melting point and mass spectral data are provided here for the first time. To a 6 dram vial were added: (-)-2-hydroxy-*N*-n-propyl-10, 11-propylidenenorapomorphine (**6**) (320 mg, 0.91 mmol) and 1-bromoethyl-2-(t-butyldimethyl)silyloxy ethane (**7**, 483 mg). Next, THF (10 mL) was added; and after the solids dissolved, 5 N NaOH (10 mL) was added and the solution was stirred. Next, Bu_4NI (113 mg) was added and the mixture was stirred at 85 ºC for 8 hours. An additional portion of **7** (322 mg) was added and the mixture stirred at 85 °C for another 5 hours. After cooling to RT, the mixture was diluted with water (30 mL) and extracted with CH_2Cl_2 (3 × 20 mL). The combined organic extracts were washed with brine, dried over Na_2SO_4, filtered, and concentrated. The brown residue was purified by silica gel column chromatography using 1:20 to 1:10 MeOH:CH_2Cl_2 as eluents to afford 230 mg (0.582 mmol, 64%) of **8**. Note: the TBS ether hydrolyzed upon contact with silica gel. M.P. = 93-95 °C.

^{1}H NMR (400 MHz, Chloroform-*d*) δ 7.53 (d, *J* = 2.6 Hz, 1H), 6.70 (d, *J* = 7.8 Hz, 1H), 6.65 – 6.58 (m, 2H), 4.13 – 4.08 (m, 2H), 3.97 (t, *J* = 4.5 Hz, 2H), 3.41 (dd, *J* = 13.2, 4.0 Hz, 1H), 3.14 (ddd, *J* = 34.2, 12.6, 5.0 Hz, 3H), 2.91 (ddd, *J* = 13.0, 9.9, 6.2 Hz, 1H), 2.75 – 2.66 (m, 1H), 2.61 – 2.40 (m, 3H), 1.80 (s, 3H), 1.67 (s, 3H), 1.65 – 1.49 (m, 2H), 0.97 (t, *J* = 7.3 Hz, 3H).

^{13}C NMR (101 MHz, Chloroform-*d*) δ 157.00, 146.75, 143.97, 134.93, 132.15, 129.26, 120.01, 117.90, 117.12, 112.71, 112.00, 106.99, 77.21, 69.12, 61.55, 59.52, 56.30, 49.37, 34.34, 29.64, 26.20, 25.96, 19.38, 12.09.

HR-MS $[M+H]^+$: calcd. 396.2175; found 396.2175.

(-)-2-(4-toluenesulfonyl)oxyethoxy-N-n-propyl-10,11-propylidenenorapomorphine (MCL-556; 3)

The following synthesis is based on the protocol previously reported Finnema *et al.;*[5] however, the NMR and mass spectral data for the product are provided here for the first time. To a 5 mL vial under nitrogen were added (-)-2-hydroxyethoxy-N-n-propyl-10,11-propylidenenorapomorphine (**8**, 56.5 mg, 0.142 mmol), DMAP (1 crystal), and tosic anhydride (70 mg, 0.214 mmol). Next, anhydrous dichloromethane (2 mL) was added, followed by trimethylamine (61 µL, 0.44 mmol), and the reaction was allowed to stir overnight at RT. The reaction was quenched by diluting with dichloromethane (5 ml) and washing sequentially with water (2 × 10 mL) and brine (1 × 10 mL). The organic layer was dried over Na_2SO_4, then filtered and concentrated *in vacuo*. The residue was purified by silica gel column chromatography using CH_2Cl_2 in a 1:50 to 1:20 (MeOH:CH_2Cl_2) gradient to afford compound **3** in 29% yield (23 mg, 0.041 mmol).

^{1}H NMR (300 MHz, Chloroform-*d*) δ 7.82 (d, *J* = 8.3 Hz, 2H), 7.48 (d, *J* = 2.6 Hz, 1H), 7.39 – 7.29 (m, 2H), 6.71 (d, *J* = 8.1 Hz, 1H), 6.63 (d, *J* = 7.7 Hz, 1H), 6.49 (d, *J* = 2.6 Hz, 1H), 4.38 (t, *J* = 4.8 Hz, 2H), 4.18 (td, *J* = 4.5, 2.0 Hz, 2H), 3.37 (t, *J* = 6.9 Hz, 2H), 3.21 – 2.96 (m, 2H), 2.76 (d, *J* = 15.9 Hz, 4H), 2.42 (s, 3H), 1.81 (s, 3H), 1.79 – 1.70 (m, 3H), 1.68 (s, 3H), 1.01 (t, *J* = 7.3 Hz, 3H).

^{13}C NMR (75 MHz, $CDCl_3$) δ 156.90, 146.96, 144.92, 144.00, 133.43, 132.68, 132.31, 129.82 (2C), 127.91 (2C), 127.51, 120.24, 118.19, 116.40, 112.73, 112.50, 107.34, 68.06, 65.43, 59.59, 55.01, 48.92, 33.06, 29.61, 27.84, 26.13, 25.87, 21.59, 18.29, 11.79.

HR-MS $[M+H]^+$: calcd. 550.2263; found 550.2272.

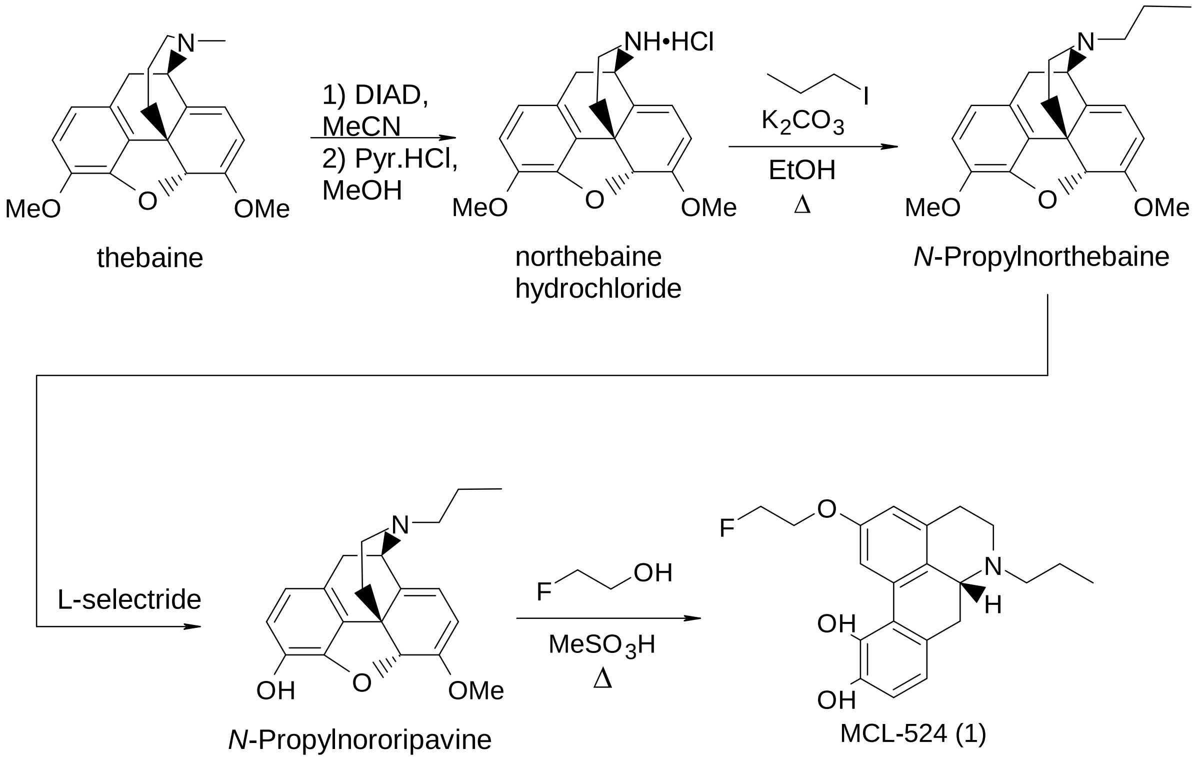

Scheme S2. Synthesis of MCL-524 (1)

Details related to the synthesis of MCL-524 (1)

(-)-2-(fluoroethoxy-N-n-propylnorapomorphine (MCL-524; 1), hydrochloride salt

MCL-524 (HCl salt) was prepared in two steps from R-(-)-*N*-n-propylnorthebaine as described in Si *et al.*[2] and Sromek, *et al.*[6] Overall yield: 20 %. M.P. = 175 °C (decomposed). Lit. 203-205 °C (decomposed).[6]

^{1}H NMR (HCl salt, 300 MHz, CD_3OD) δ 8.13 (d, *J* = 2.5 Hz, 1H), 6.84 – 6.73 (m, 3H), 4.83 (t, *J* = 4.0 Hz, 1H), 4.68 (t, *J* = 4.0 Hz, 1H), 4.38 – 4.28 (m, 2H), 4.26 – 4.20 (m, 1H), 3.71 – 3.53 (m, 1H), 3.47 – 3.35 (m, 3H), 3.30 – 3.04 (m, 3H), 2.79 (t, *J* = 13.5 Hz, 1H), 2.07 – 1.72 (m, 2H), 1.22 – 1.09 (m, 6H). 1.22 – 1.09 (m, 3H).

^{13}C NMR (75 MHz, CD_3OD) δ 159.73, 146.40, 144.90, 135.73, 131.98, 125.66, 122.22, 120.82, 119.99, 115.96, 115.29, 113.19, 83.12 (d, *J* = 169.1 Hz), 68.63 (d, *J* = 19.8 Hz), 62.55, 56.59, 32.84, 27.48, 25.25, 18.36, 11.28. ^{19}F NMR (282 MHz, CD_3OD) δ 4.20 (tt, *J* = 47.7, 28.9 Hz).

^{19}F NMR (376 MHz, Methanol-d_4) δ -218.80 (tt, *J* = 47.2, 23.2 Hz).

HR-MS $[M+H]^+$: calcd. 358.1818; found 358.1826.

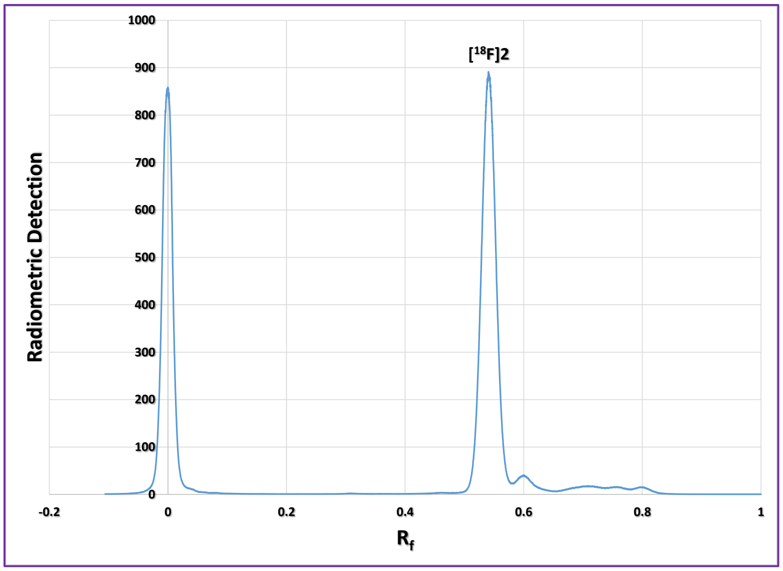

Figure S1. Representative radio-TLC of intermediate [^{18}F]2. Reaction conditions: precursor **3** (1.1 mg), TEATos (2.4 mg), 97% MeCN (1 mL), 150 °C, 15 min). Radiochemical conversion = 53%. Eluent = 10% MeOH in CH_2Cl_2.

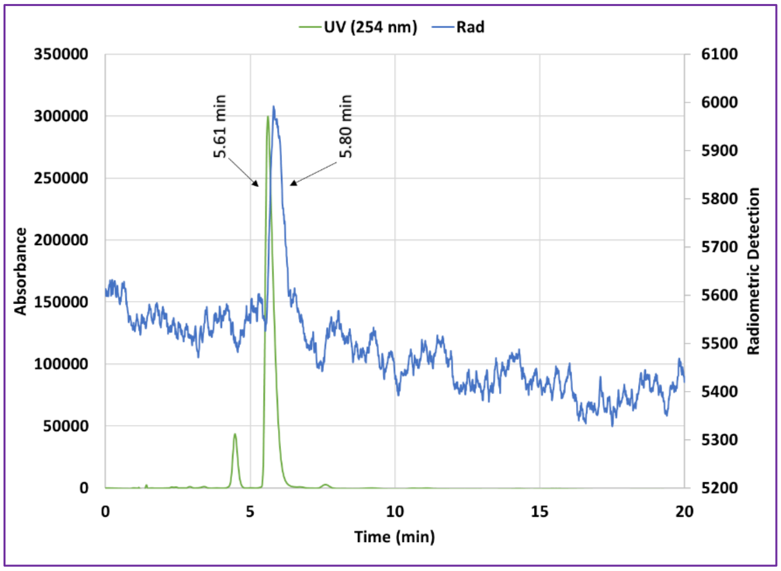

Figure S2. Co-injection of [^{18}F]MCL-524 with ^{19}F standard. HPLC 1, Program C.

References:

[1] Kim, K.; Kim, K. Synthesis of the new thebaine derivatives by the Diels-Alder reaction with northebaine. *J. Korean Chem. Soc.* **1988,** *32* (4), 371-376

[2] Si, Y. G.; Gardner, M. P.; Tarazi, F. I.; Baldessarini, R. J.; Neumeyer, J. L. Synthesis and dopamine receptor affinities of N-alkyl-11-hydroxy-2-methoxynoraporphines: N-alkyl substituents determine D_1 versus D_2 receptor selectivity *J. Med. Chem.* **2008**, *51*, 983-987.

[3] Gao, Y. G.; Baldessarini, R. J.; Kula, N. S.; Neumeyer, J. L. Synthesis and dopamine receptor affinities of enantiomers of 2-substituted apomorphines and their N-n-propyl analogues. *J. Med. Chem.* **1990,** *33* (6), 1800-1805.

[4] Steiger, C.; Finnema, S. J.; Raus, L.; Schou, M.; Nakao, R.; Suzuki, K.; Pike, V. W.; Wikström, H. V.; Halldin, C., A two-step one-pot radiosynthesis of the potent dopamine D2/D3 agonist PET radioligand [^{11}C]MNPA. *J. Labelled Compd. Radiopharm.* **2009,** *52* (5), 158-165.

[5] Finnema, S. J.; Stepanov, V.; Nakao, R.; Sromek, A. W.; Zhang, T.; Neumeyer, J. L.; George, S. R.; Seeman, P.; Stabin, M. G.; Jonsson, C.; Farde, L.; Halldin, C. ^{18}F-MCL-524, an ^{18}F-Labeled Dopamine D_2 and D_3 Receptor Agonist Sensitive to Dopamine: A Preliminary PET Study. *J. Nucl. Med.* **2014,** *55* (7), 1164-1170.

[6] Sromek, A. W.; Si, Y. G.; Zhang, T.; George, S. R.; Seeman, P.; Neumeyer, J. L. Synthesis and Evaluation of Fluorinated Aporphines: Potential Positron Emission Tomography Ligands for D_2 Receptors. *ACS Med. Chem. Lett.* **2011,** *2* (3), 189-194.
